# Fungal Endophytes to Combat Biotic and Abiotic Stresses for Climate-Smart and Sustainable Agriculture

**DOI:** 10.3389/fpls.2022.953836

**Published:** 2022-07-05

**Authors:** Anamika Verma, Nowsheen Shameem, Hanuman Singh Jatav, Eetela Sathyanarayana, Javid A. Parray, Peter Poczai, R. Z. Sayyed

**Affiliations:** ^1^Amity Institute of Horticulture Studies and Research, Amity University Uttar Pradesh, Noida, India; ^2^Department of Environmental Science, S.P. College, Srinagar, India; ^3^Department of Soil Science and Agricultural Chemistry, Sri Karan Narendra Agriculture University, Jaipur, India; ^4^Agricultural College-Palem, PJTSAU, Hyderabad, India; ^5^Department of Environmental Science, Government Degree College Eidgah, Srinagar, India; ^6^Finnish Museum of Natural History, University of Helsinki, Helsinki, Finland; ^7^Department of Microbiology, PSGVP Mandal’s SI Patil Arts, GB Patel Science and STKV Sangh Commerce College, Shahada, India

**Keywords:** abiotic stress, biotic stress, climate-resilience, drought, fungal endophytes, sustainable agriculture

## Abstract

The agricultural sustainability concept considers higher food production combating biotic and abiotic stresses, socio-economic well-being, and environmental conservation. On the contrary, global warming-led climatic changes have appalling consequences on agriculture, generating shifting rainfall patterns, high temperature, CO_2_, drought, etc., prompting abiotic stress conditions for plants. Such stresses abandon the plants to thrive, demoting food productivity and ultimately hampering food security. Though environmental issues are natural and cannot be regulated, plants can still be enabled to endure these abnormal abiotic conditions, reinforcing the stress resilience in an eco-friendly fashion by incorporating fungal endophytes. Endophytic fungi are a group of subtle, non-pathogenic microorganisms establishing a mutualistic association with diverse plant species. Their varied association with the host plant under dynamic environments boosts the endogenic tolerance mechanism of the host plant against various stresses *via* overall modulations of local and systemic mechanisms accompanied by higher antioxidants secretion, ample enough to scavenge Reactive Oxygen Species (ROS) hence, coping over-expression of defensive redox regulatory system of host plant as an aversion to stressed condition. They are also reported to ameliorate plants toward biotic stress mitigation and elevate phytohormone levels forging them worthy enough to be used as biocontrol agents and as biofertilizers against various pathogens, promoting crop improvement and soil improvement, respectively. This review summarizes the present-day conception of the endophytic fungi, their diversity in various crops, and the molecular mechanism behind abiotic and biotic resistance prompting climate-resilient aided sustainable agriculture.

## Introduction

Sustainable agriculture goals provide a new framework to consider climate change within multiple dimensions of sustainability, considering it as one of the major hindrances in achieving food security for an ever-increasing population. According to IPCC (2014) report, the global temperature may rise from 1.7 to 4.8°C, altering precipitation patterns by the twenty-first century. Abiotic stress driven by climatic changes has reduced global agricultural production by 1 to 5% during the last three decades elevating global food demand from 70 to 100% by 2050 (Newbery et al., 2016; Pais et al., 2020). Climate change has a risk on the ecosystem. Its adaptation is necessary as it imparts short and long-term effects on food production and concurrently displays a significant impact on agriculture, primarily through greenhouse gas emissions (Pais et al., 2020). According to IPCC (2018) report, cumulative emission of carbon dioxide (CO_2_) and future non-CO_2_ radiative forcing determine the probability of limiting global warming impacts of 1.5°C. The dire effect of climate change can be observed on agricultural productivity affecting overall sustainable agricultural systems. Various abiotic stress conditions, such as high carbon dioxide, drought, high temperature, salinity, etc., hamper food production (Keswani et al., 2019a). Generation of ROS during stress such as singlet oxygen (^1^O_2_), hydrogen peroxide (H_2_O_2_), superoxide radical (O_2_), hydroxyl radical (OH^–^), etc., decreases crop yield (Keswani et al., 2019a). Thus sustainable agriculture needs to be climate-resilient agriculture at the first place. Various modern techniques like breeding, biotechnology, screening techniques, land reformation, etc., should be incorporated altogether, and new possibilities must be explored and given a chance.

On the other hand, over-reliance on synthetic pesticides in the era of the green revolution has anticipated adverse environmental impacts generating pesticide resistance, insect resurgence, effects on non-target organisms, environmental pollution, etc., demanding a lasting conservational approach toward plant protection (Meena et al., 2020; Jacquet et al., 2022). Crops are infested by various pests and pathogens (like fungi, bacteria, viruses, nematodes, etc.), which need a comprehensive antidote to overthrow food production and environmental conservation-related insecurities. Alternative tactics like biological control/biocontrol agents utilizing microorganisms for plant protection seem more reliable and ecosystem-friendly (Adeleke et al., 2022). Critical plant-associated microorganisms like endophytes can play a pivotal role in alleviating the response of crops toward climate change (Yan et al., 2019; Grabka et al., 2022). Fungal endophytes are most studied among other endophytes. Endophytic fungi are an ecological, non-pathogenic, polyphyletic group of highly diverse fungi of ascomycetes, basidiomycetes, and anamorphic fungi (Gupta et al., 2020). They may enter the tissue of their hosts, colonize them, and offer affirmative impacts (Ray et al., 2017). Fungal endophytic colonization induces physiological changes and modifies gene expression in the plants, thereby uplifting plant productivity *via* higher photosynthesis rate, promoting the shoots and roots growth, enhancing uptake and nutrient use efficiency conferring abiotic (drought, salinity, high temperature, high CO_2_, and metal toxicity) and biotic stress (pathogens and pests) tolerance (Harman and Uphoff, 2019; Grabka et al., 2022; Liu-Xu et al., 2022). Many fungal endophytes have been reported and extracted from healthy plants potent enough to be incorporated artificially as biofertilizers and biocontrol agents in crops (Busby et al., 2016; Bastami et al., 2021; Grabka et al., 2022). Vis-à-vis application of various biofertilizers are also reported to enhance fungal endophyte colonization (Kumar et al., 2021). Endophytic fungi have also been efficient in environmental remediation (agrochemical and metal pollutants solubilization/bio assimilation/mineralization) (Gavrilaş et al., 2022). They can also be utilized as a nanosensor for detecting the contaminants, which, if further explored and incorporated, could help in environmental conservation (Sahoo et al., 2017; Khanam et al., 2020). However, environmental remediation is a broad department involving time-consuming processes and demands intensive research to resolve all associated risk factors. Henceforth, the present review aims to explore the role of symbiotic fungal endophytes, their interactions, and the mechanism involved in conferring adaptation to crops against abiotic and biotic stressed conditions, which may contribute toward climate resilience and aiding sustainable agriculture.

## Endophytic Fungi: Treaty of Amity

**FIGURE 1:**
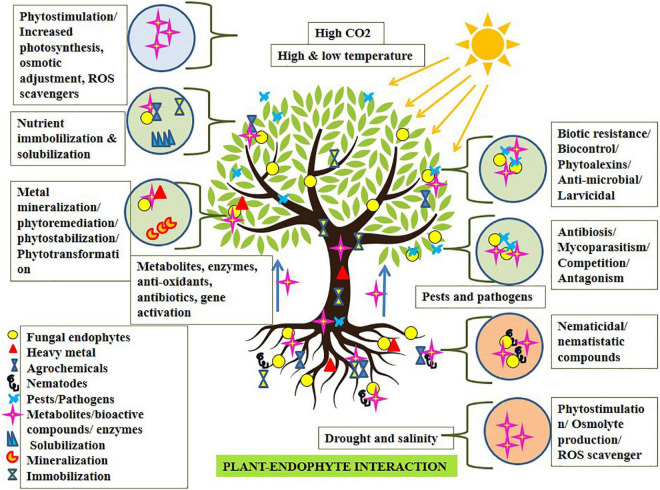
Fungal endophyte-plant interaction expediting phytostimulation conferring stress mitigation.

Broad-spectrum endophytic fungi follow direct or indirect mechanisms for benefiting the plants. The direct beneficial mechanism involves phytohormone production, nitrogen fixation, phosphate solubilization, siderophore production, and anti-microbial metabolite production (El Enshasy et al., 2020). The indirect beneficial mechanism involves abiotic and biotic resistance (modifying the metabolism process), biocontrol, bioremediation, and phytoremediation (Dubey et al., 2020; Singh et al., 2021). Cosmopolitan fungal endophytes act as biostimulants to produce certain bioactive compounds, phytohormones, phosphate solubilization factors, etc., to enhance root growth, seed germination, and plant growth promotion (Rustamova et al., 2022). Fungal endophytes may potentially execute bounteous ubiquitous roles in the host plant *via* phytostimulation, phytoimmobilization, phytostabilization, phytotransformation, phytoremediation, biocontrol, etc. (Sahoo et al., 2017; Radziemska et al., 2021; Adeleke et al., 2022; Figure 1). They are reported to produce secondary metabolites which include bioactive anti-microbial siderophores which may execute defense against various pathogens (Srinivas et al., 2020). The endophytes associated with plants produce hydroxamate and carboxylate type siderophores of several classes which play a significant role in transporting iron and zinc to the plants. Siderophores solubilize an array of metals like cadmium, molybdenum, manganese, zinc, nickel, copper, and actinides and are found to mobilize them and make them available to the plants (Rana et al., 2020). To date, more than 500 siderophores have been documented from various fungi (Ahmed and Holmström, 2014; Chowdappa et al., 2020). Recently, many fungal endophytes have been identified which solubilize and mobilize phosphorus (*Penicillium*, *Aspergillus*, *Curvularia*, *Trichoderma*, *Mesorhizobium*, etc.) (Mehta et al., 2019), potassium (*Aspergillus fumigatus* and *A. niger*) (Haro and Benito, 2019), and zinc salts (*Alternaria thlaspis* and *Metapochonia rubescens*) (Yung et al., 2021), boosting plant metabolic activity, plant growth resulting in high crop production. They execute phytostimulation *via* lowering plant hormone ethylene levels by 1-aminocyclopropane-1-carboxylate deaminase (ACC), escalating plant growth (Nascimento et al., 2014; Singh et al., 2015); they may biodegrade biomass and recycle them in the environment, enhancing nitrogen availability, Zinc and phosphorus uptake for the host resulting in phytoimmobilization (Yung et al., 2021). Phytoimmobilization ultimately boosts plants to sustain abiotic stresses *via* immobilizing osmolytes and stabilizing membrane ion conductivity during stress conditions. Phytotransformation is the neutralization of soil pollutants, for example, pesticides, industrial by-products, xenobiotics, etc. Endophytic fungi like *Piriformospora* sp., *Exophiala* sp., *Neotyphodium* sp., etc., associated with many crop species, are reported to remediate soil from metal pollutants, avoiding phytotoxicity (Ningxiao et al., 2016; Sahoo et al., 2017). However, bioremediation utilizing fungal endophytes demands focused study in the near future incorporating endophyte-led nanosensors to unveil its potential in environmental conservation (Khanam et al., 2020). Finally, biofertilizers for plant-growth promotion and biocontrol agents for mitigation of pests and diseases is its other major operation. In this context, culture-independent molecular techniques using metagenomics and multi-locus sequence typing can help isolate and characterize such endophytes (Gupta et al., 2020; Adeleke et al., 2022; Chouhan et al., 2022). Wherein species-specific fungal endophytes identification, isolation, characterization, inoculation, and interaction studies, unraveling their molecular signaling mechanism, and delivering abiotic and biotic stress mitigation, are highly expected. Furthermore, not only isolation but studies related to the shelf-life enhancing components for such fungal biocontrol agents and biofertilizers (Zope et al., 2019) are imperative.

### Biodiversity of Fungal Endophytes

The biodiversity of fungal endophytes is vast and reported from almost all plants/crops worldwide (Suryanarayanan et al., 2018; Table 1). According to Rodriguez et al. (2009), fungal endophytes can be classified into four groups based on the parts they colonize and their transmittance- (a) Class I are Clavicipitaceous fungi (resides in grasses with vertical transmittance), (b) Class- II-IV are non-clavicipitaceous fungi (Ascomycota and Basidiomycota), wherein Class-II endophytes inhabits roots, shoots, and rhizomes, and transmit through seeds or rhizomes (i.e., horizontally and vertically both), (c) Class-III inhabits leaves/shoots (transmitted horizontally) and (d) class-IV are typical ascomycetes, forming conidia and resides in roots (transmitted horizontally) of the plants. Class, I endophytes are free-living symbiotic species associated with grasses, rushes, and sedges. Their associations help plants in nematode resistance, anti-fungal compound production, and abiotic stress mitigation, including drought and metal tolerance (*Epichloe festucae*, *Neotyphodium tembladerae*, etc.). Class II endophytes increase plant root and shoot biomass and confer tolerance against disease, drought, desiccation heat, and salinity (for example, *Mycophycia ascophylli*, *Phoma* sp., *Arthrobotrys* sp., etc.). Class III includes hyper-diverse fungal endophytes mostly inhabiting above-ground tissues and isn’t habitat-specific. They motivate the interaction among various microorganisms’ populations (for example, *Ustilago maydis*, *Phyllosticta* sp., *Colletotrichum* sp., etc.) (Chitnis et al., 2020). Class IV endophytes are root-associated sterile ascomycetous fungi forming melanized structures prevalent in high-stress environments worldwide (for example, *Chloridium paucisporum*, *Phialocephala dimorphosphora*). Overall, most of the fungal endophytes reported in crops belong to Ascomycota and Basidiomycota (Rodriguez et al., 2009). These endophytes produce secondary metabolites that confer host plants resistance (Table 2). The secondary metabolites produced may be natural like phytohormones or any nutrients or alkaloids, terpenes, quinines, benzopyranones, chinones, phenolic acids, terpenoids, steroids, flavonoids, hydrocarbons, etc., in response to stressed condition (Tiwari, 2015; Bilal et al., 2018; Chouhan et al., 2022); depending upon fungal endophyte and host species (Grabka et al., 2022). Therefore, it is essential to gain in-depth knowledge of their host range, specificity, colonization, transmission, and host-endophyte interaction.

**TABLE 1:** Fungal endophytes recently reported in important agronomic and horticultural crops grown worldwide.

Crop	Botanical name	Naturally occurring fungal endophytes	References
Wheat	*Triticum aestivum*	*Alternaria, Acremonium, Aureobasidium, Cladosporium, Penicillium, Sarocladium, Anthracocystis, Cryptococcus, Sporobolomyces, Vishniacozyma*	Rojas et al., 2020
Apple	*Malus domestica*	*Chaetomium, Epicoccum, Biscogniauxia, Penicillium, Diaporthe, Phlyctema*	Liu et al., 2020
Orchid	*Vanda cristata*	*Fusarium sp.*	Srivastava et al., 2018; Chand et al., 2020
Crucifers	*Brassica oleracea, B. rapa and Raphanus sativus*	*Colletotrichum, Fusarium, Cladosporium, Trichoderma* (major) *Aspergillus bombycis, Aspergillus nomius, Aspergillus penicillioides, Aspergillus tamarii, Aspergillus westerdijkiae, Cladosporium subuliforme, Cladosporium xanthochromaticum, Trichoderma hamatum, T. harzianum, and Zopfiella* (stems) *Alternaria alternata, Alternaria burnsii, Phialemoniopsis pluriloculosa, Aspergillus flavipes, Colletotrichum siamense, F. fujikuroi, Gilmaniella subornata, Macrophomina sp., Mycotribulus mirabilis and Penicillium citrinoviride* (leaves)	Chen et al., 2020
Onion	*Allium cepa*	*Clonostachys rosea, Fusarium sp., Hypocrea lixii, Trichoderma asperellum, T. atroviride, T. harzianum*	Muvea et al., 2014; Caruso et al., 2020
Chicory	*Cichorium intybus*	*Cladosporium, Epicoccum, Septoria, Plectosphaerella, Alternaria*	Hatamzadeh et al., 2020
Citrus	*Citrus reticulata*	*Alternaria alternate, Alternaria brassicicola, Alternaria carthami, Ascochyta medicaginicola, Aspergillus pallidofulvus, Aureobasidium melanogenum, Cladosporium cladosporioides, Colletotrichum, Diaporthe, Didymella, Fusarium, Meyerozyma, Myrothecium, Neocosmospora, Neosetophoma, Phaeoacremonium, Pseudozyma, Scedosporium, Talaromyces*	Sadeghi et al., 2019
Chives	*Allium schoenoprasum*	*Beauveria brassiana, Penicillum pinophilum*	Espinoza et al., 2019
Tomato	*Solanum lycopersicum*	*Alternaria, Aspergillus, Chaetomium, Curvularia, Fusarium, Hypoxylon, Leptosphaerulina, Meyerozyma, Nigrospora, Penicillium, Periconia, Stemphyllium, Trichoderma*	Bogner et al., 2016; Xia et al., 2019
Chrysanthemum	*Dendrobium sp.*	*Fusarium sp.*, *Colletotrichum* sp.	Shah et al., 2018
Neem	*Azadirachta indica*	*Chloridium*, *Colletotrichum, Curvularia, Fusarium, Trichoderma, Penicillium, Phyllostica, Phoma, Verticillium, Xylaria*	Chutulo and Chalannavar, 2018
Banana	*Musa paradisica*	*Colletotrichum, Cochliobolus, Fusarium, Lasiodiplodia, Nigrospora, Pestalotiopsis, Phoma, Penicillium*	Zakaria and Aziz, 2018
Maize	*Zea mays*	*Acremonium zeae, Cladosporium oxysporum, Colletotrichum boninense, Colletotrichum gloeosporioides, Coprinopsis cinerea, Curvularia lunata, Epicoccum sorghinum, Fusarium fujikuroi, Gibberella moniliformis, Nemania* sp*., Penicillium* sp., *Rigidoporus vinctus, Sarocladium zeae, Scopulariopsis gracilis*.	Renuka and Ramanujam, 2016
Onion	*Allium longicuspis*	*Alternaria* sp*., A. terreus, Aspergillus ochraceus, Aspergillus versicolor, Aspergillus spectabilis, A. flavus, Fusarium sambucinum*	Abdulmyanova et al., 2016
Common bean	*Phaseolus vulgaris*	*Aureobasidium pullulans, Fusarium oxysporum, Xylaria* sp., *Cladosporium cladosporioides*	Parsa et al., 2016
Periwinkle	*Catharanthus roseus*	*Colletotrichum, Fusarium, Macrophomina, Nigrospora*	Ayob and Simarani, 2016
Lemon grass	*Cymbopogon citratus*	*Colletotrichum, Fusarium, Penicillium, Phoma*	Chow and Ting, 2015
Curry tree	*Murraya Koenigii*	*Colletotrichum, Fusarium, Penicillium, Phoma*	Chow and Ting, 2015
Rapeseed	*Brassica napus*	*Alternaria, Aspergillus, Botrytis, Cryptococcus, Epicoccum, Fusarium, Penicillium, Rhizoctonia, Phoma, Rhizopus, Sporobolomyces*, etc.	Zhang et al., 2014
Tea plant	*Camellia sinensis*	*Colletotrichum gloeosporioides*	Rabha et al., 2014
Wood sorrel	*Oxalis corniculata*	Aspergillus, Chladosporium, Cunninghamella, *Fusarium, Rhizopus*	Kurandawad and Lakshman, 2014

**TABLE 2:** Endophytic fungi and their wide range of bioactive compounds with their properties.

Endophytic fungus/fungi	Bioactive compounds/metabolites	Chemical classes/group	Properties	References
*Alternaria* sp.	Altersolanol A (hydroxylated quinone); Altenusin	Quinones; alkaloids	Anti-bacterial	Mousa and Raizada, 2013; Anamika et al., 2018
*Aspergillus* sp.	Cyclopeptides echinocandins; indoloditerpenes and asporyzin	Peptides; Sesquiterpenes (Diterpenes)	Anti-fungal; pesticidal activity	Mousa and Raizada, 2013; Anamika et al., 2018
*Pezicula* sp.	(R)-mellein; Cyclopeptides echinocandins	Isocaumarin derivative; peptides	Anti-microbial; larvicidal	Anamika et al., 2018
*Neotyphodium* sp.	Peramine; agroclavine, chanoclavine and elymoclavine; ergot alkaloids; tricin	Pyrrolopyrazine alkaloid; indole alkaloids; flavanoids	Pesticidal; anti-microbial; larvicidal	Mousa and Raizada, 2013; Anamika et al., 2018
*Penicillium* sp.	Volatile organic compounds; Penicisteroid A	VOCs; Steroids	Promotes growth and starch accumulation; anti-fungal property	Mousa and Raizada, 2013; Anamika et al., 2018
*Phyllostica sp.*	Hydroheptelidi, heptelidic acid; chlorinated metabolites	Sesquiterpenes; chlorinated compounds	Larvicidal	Anamika et al., 2018
*Phomopsis* sp.	Phomopsichalasin; Cycloepoxylactone and cycloepoxytriol B	Amine and amide alkaloids; sesquiterpene	Anti-bacterial; pesticidal activity	Mousa and Raizada, 2013; Anamika et al., 2018
*Acremonium* sp.	Leucinostatin A; Peramine (loline alkaloids); pyrrocidines A and B	Oligopeptide; amines and amides; polyketide amino acid	Phytotoxic; anti-fungal; anti-feedants; antibiotics	Mousa and Raizada, 2013; Anamika et al., 2018
*Cladosporium* sp.	Methyl benzoate; 2-Methoxy-4-hydroxy-6-methoxymethylbenzaldehyde	Volatile compounds; phenols	Anti-microbial; anti-fungal	Anamika et al., 2018
*Colletotrichum* sp.	6-isoprenylindole-3-carboxylic acid and steroids; Colletotric acid	Indole alkaloids and steroids; phenol	Anti-fungal, anti-bacterial, antagonistic and pesticidal	Anamika et al., 2018
*Trichoderma viride*	Isobutyl alcohol, isopentyl alcohol, 3-methylbutanal, α-bergamotene, bicyclogermacrene, farnesene, geranylacetone, β-sesquiphellandrene, valencence, α-ylangene and zingiberene	Volatile organic compounds	Promotes plant growth, development and flowering	Anamika et al., 2018
*Fusarium* sp.	Diterpene subglutinol; β-caryophyllene	Terpenoids (diterpenes); VOCS	Anti-microbial; growth promoter	Anamika et al., 2018
*Beauveria* sp.	Chitinases, lipases and proteases; virulence factor	Enzymes and virulence factor	Pesticidal property	Anamika et al., 2018
*Phoma* sp.	1-N-methylalbonoursin; Altersolanol A; 2-hydroxy-6-methylbenzoic acid; Phomadecalin C	Amine and amide alkaloids; Quinone; phenol; sesquiterpenes	Anti-microbial; antagonistic effect	Anamika et al., 2018
*Ampelomyces* sp.	Altersonalol A	Tetrahydroanthraquinones	Biocontrol agent of parasitic fungus	Mousa and Raizada, 2013
*Trichoderma harzianum*	Trichodermin	Sesquiterpenes	PGR synthesis, anti-fungal and inhibitory	Mousa and Raizada, 2013
*Taxomyces andreanae*	Paclitaxel (Taxol)	Sesquiterpenes (Diterpenes)	Anti-fungal property	Mousa and Raizada, 2013
*Xylaria* sp.	Eremophilane sesquiterpene; sordarin	Pheromones; Sesquiterpenes (Diterpenes)	Anti-fungal; phytotoxic	Mousa and Raizada, 2013

## Climate-Smart Agriculture: Facts and Faiths

**FIGURE 2:**
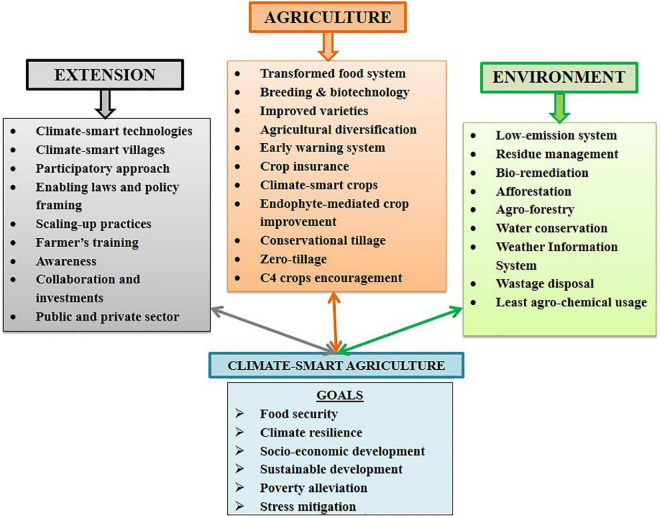
Various components succeeding CSA-led sustainable agriculture goals.

Climate-smart agriculture (CSA) is directly linked to socio-economic well-being, sustainable agroecosystem productivity, poverty alleviation, nutritional security, adaptability, and stress mitigation. CSA is anticipated by strategies such as increased resilience toward extreme stressed environments, transforming agriculture and food systems, policy formulations, and awareness promoting ecosystem services (Steenwerth et al., 2014; Fusco et al., 2020; Figure 2). Keeping the above points in mind, beneficial symbiotic fungal endophytes working at a micro-ecosystem level forming rhizosphere offer an immense scope of research for CSA benefaction. Their non-pathogenic nature, universal occurrence, triggered nutrient uptake, mineral solubilization, phytoremediation, enhanced vegetative and reproductive growth, stress tolerance resulting in optimum to high yield, etc., make them suitable for organizing full-fledged scientific research and attention (Galani et al., 2022). Fungal endophytes are reported to increase biomass and remove litter from the soil, increase mineral status and nutrient uptake which aids in achieving CSA concept indirectly (Datta et al., 2017; Santamaria et al., 2017). Whereby, litter is a soil organic matter with a complex structure and a recalcitrant nature that is difficult to degrade wherein, endophytic microbes can be helpful. Fungal endophyte-mediated crop improvement can bring sea changes. However, it suffers from some associated bottlenecks, for example, colonization of a new host plant with a suitable endophyte, their symbiotic association and native endobiome (their direct and indirect effect) (Keswani et al., 2019b). The association is further influenced by their genetics, gene expression, molecular patterns, behavior alteration, etc. Thus, better insight into the behavior of an introduced endophyte and its beneficial/harmful association with host plant for long term if any, and to what extent is inevitable (Chitnis et al., 2020). Other barriers include economic barriers (high initial cost, high implementation cost, return uncertainty, etc.), institutional and organizational barriers (low support, lack of regulatory framework and policies, etc.), ease of conventional methods, and market barriers (lack of awareness, lack of management and poor market, etc.) impede its overall adoption (Fusco et al., 2020). Consequently, comprehensive and collaborative investments indulging long-term commitment from scientific and researcher communities, farmers, social communities, environmentalists, and policymakers are mandatory to fuel the prolonged CSA concept serving sustainability.

## Fungal Endophytes for Abiotic Stress Management

**FIGURE 3:**
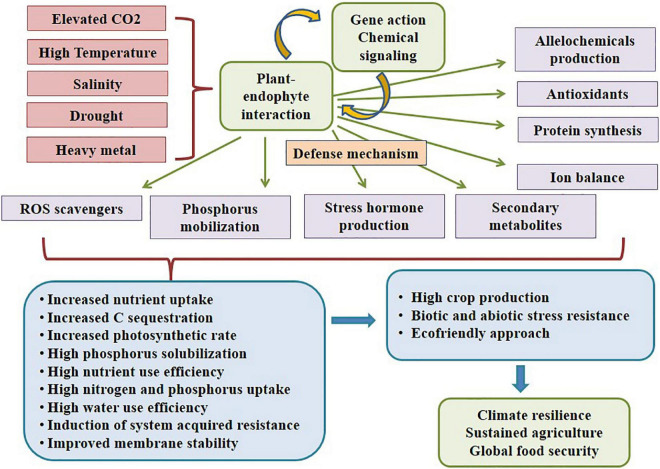
Plant-endophyte symbiotic interaction conferring abiotic resistance.

Plant growth is greatly hampered by abiotic stresses, and to tolerate it; the plant needs a proficient system or mechanism. Usually, several microbes are found to acquire nutrients from various plants, where some interactions are beneficial and some are harmful to the host. According to Ripa et al. (2019), microbes that inhabit the plant tissues devoid of doing substantive damage or acquiring remuneration other than securing their residency are considered ‘endophytes.’ Every plant harbors one or a few endophytes that supply nutrients, enhance environment acclimatization, and confer biotic and abiotic stress tolerance in the host plant. An endophytic fungus lives in inter or intracellular spaces in the stem, petiole, and leaves of a plant for a part or whole of its life cycle. These highly efficient fungal endophytes can ensue for the entire life of the plant, which might also enable the plant to squelch the environmental constraints (Vandenkoornhuyse et al., 2015; Chouhan et al., 2022). Fungal endophytes are crucial for resilience and sustainability in today’s agriculture. They offer diverse applications in the agricultural system by collaborating with host plants (Redman et al., 2021; Grabka et al., 2022), as depicted in Figure 3. They possess a vital ability to mobilize insoluble phosphate and provide nitrogen to their host plants boosting vegetative growth sequentially and strengthening associated plants to confer resistance (Hassan, 2017; Yadav and Yadav, 2017). During stress, the fungal endophytes secrete an alleviated amount of plant growth-promoting metabolites; for example, *Penicillium* sp. inhabiting *Suaeda japonica* secretes phytohormone Gibberellins. Likewise, *Aspergillus* sp., *Cladosporium* sp., *Fusarium* sp., *Penicillium* sp., *Verticillium* sp., and Ascomycete sp. inhabiting *Panax ginseng* secretes triterpenoid saponins and ginsenosides (enhances root growth and biotic and abiotic stress tolerance) (Sahoo et al., 2017). Keswani et al. (2022) reviewed biosynthesis of giberellins from various fungi imparting beneficial effects on the crop production. Similarly, *Phoma* sp. inhabiting *Tinospora cordifolia* secretes growth hormones; *Trichoderma atroviride*, *T. polysporum*, and *T*. *harzianum* inhabiting *Phaseolus vulgaris* secrete proteolytic enzymes, phosphate solubilizing factors, active volatile, and non-volatile metabolites, and *Mucor* sp. inhabiting *Brassica campestris* enhances Indole-3 amino acid (IAA), ACC deaminase and solubilize phosphate secretion resulting in plant growth promotion (Sahoo et al., 2017). Fungal endophytes perform phosphate solubilization, siderophores production, antibiotic production, phytohormone production, nutrient mineralization, etc., functions for plant growth promotion during stress conditions (Yung et al., 2021). Due to abiotic stress stimuli, abscisic acid, jasmonic acid, and salicylic acid are produced and act as defense signaling substances. Most soil types are insufficient in phosphorus content, where P is considered an essential element for plant growth. They remain insoluble and unavailable to plant even when applied as an inorganic fertilizer to the soil; hence, aluminum and iron hydroxides commence their fixation in acidic soil. Iron having redox activity behaves as a cofactor of many enzymes and is crucial as a micronutrient but remains in insoluble (ferric hydroxide) form in the soil (Johnson, 2008). Fungal endophytes produce siderophores with low molecular weight and a high affinity for iron (Fe3+), thereby increasing iron uptake and solubilization of phosphates and their uptake by the plants, promoting growth. Endophytes biodegrade the litter and enhance biomass production and nutrient uptake (Idbella et al., 2019).

### High Carbon Di-Oxide

Endophytic fungi increase internal hyphal growth and colonization, carbon sequestration, biomass, and yield *via* increased photosynthesis and induced compositional changes in elevated CO_2_ (Galani et al., 2022). In this context, various researchers found symbiotic relationship beneficial as it escalates plant growth in crops viz. tomato, rice, beans, cereals, potato, etc., secreting ascorbate and glutathione reducing Reactive Oxygen Species (ROS), following antioxidants and allelochemicals production (Yadav and Yadav, 2017). This renders efficient nutrient uptake, photosynthetic rate, water use efficiency (Elhindi et al., 2016), elevating phytohormone production, amplifying gene expression, supplementing protein function, metabolite synthesis, signaling molecules secretion, and triggering stress-responsive pathways (Ma et al., 2019), for better survival without hampering the yield. However, some contrast results revealing decreased root and shoot growth exaggerated that endophytes vary in their effects on the same host plants subjected to the specific niche, preferentially under a particular growth condition (Gavito et al., 2000). Furthermore, colonization is also affected by the type of plants; for example, C3 plants consume additional carbon for biomass production and thus are less likely to be benefited from colonization, whilst C4 plants allocate more carbohydrates resulting in a profit from endophytic fungi association (Tang et al., 2009). Therefore, according to the present studies, the role of microbiota in biological and ecological processes remains a challenge (Rehman et al., 2016), especially for possible application in agriculture as endophytes adopt a molecular mechanism to induce stress-responsive genes, anti-stress metabolites, and Reactive Oxygen Species (ROS) scavenger molecules synthesis.

### Drought

Drought is one of the significant stresses diminishing agricultural productivity and directly challenging food security attainment goals in developing countries. A range of physiological and biochemical responses are triggered in the plants to respond to drought conditions, such as stomatal closure, increased respiration, suppression of growth, and photosynthesis. According to researchers, two groups of drought stress-inducible genes function to confer plant tolerance, the first being the proteins and the second regulatory proteins (involved in signal transduction) (Shinozaki and Yamaguchi-Shinozaki, 2007). Fungal endophyte *Piriformospora indica* (family- Sebacinaceae) colonizes many plant roots, and its hyphae are present in the outer layer of the host’s roots aiding in plant growth, higher chlorophyll content, and elevated yield under drought stress conditions; such fungal endophytes led drought stress tolerance in wheat at diverse temperature regimes has been studied by Ripa et al. (2019). The fungal genus studied for plant growth parameters and stress tolerance was *Fusarium* sp., *Trichoderma* sp., *Penicillium* sp., *Aspergillus* sp., *Alternaria* sp., etc. Most of them were found to confer drought resistance to wheat. Endophytic fungus-associated plants (rice, tomato, and dune grass) have used significantly less water and increased biomass than non-symbiotic plants. The drought tolerance phenomenon may be explained by an enhanced accumulation of solutes in tissues of endophyte-infected plants compared to non-infected plants, by reduced leaf conductance and a slowdown of the transpiration stream, or due to thicker cuticle formation (Ripa et al., 2019). Characterization of *Trichoderma* sp. inhabiting *Theobroma cacao* revealed changes in gene expression patterns, implying that *Trichoderma* sp. could induce tolerance to abiotic stresses, possibly including drought (Bailey et al., 2006). Drought, heat, and salt stress affect the plant–water relationship, triggering complex plant responses, including increased osmolytes production. According to Marquez et al. (2007), the osmotic potential is determined primarily by solute potential and matrix potential; symbiotic fungi are likely to contribute to the matrix potential, which is particularly important in helping plants retain water and enhancing plant drought tolerance. Accordingly, the symbiotic fungi-associated plants consume significantly less water than non-symbiotic plants (Marquez et al., 2007).

### Salinity

Salinity is another significant abiotic stress limiting the growth and productivity of plants worldwide. Tolerance of plants to salt stress is associated with the alleviation of antioxidant enzymes, i.e., ROS scavengers including glutathione, ascorbate, and tocopherol, and the enzymes superoxide dismutases (SOD), catalases (CAT), ascorbate- or thiol-dependent peroxidases (APX), glutathione reductases (GR), dehydroascorbate reductases (DHAR) and mono-dehydroascorbate reductases (MDHAR) (Rouhier et al., 2008). They are involved in removing ROS directly (SOD, CAT, and APX) or indirectly *via* regenerating ascorbate and glutathione in the cell. For instance, *Piriformospora indica*, a root fungal endophyte, upraised biomass production in salt stress conditions by intensifying antioxidative capacity, fatty acid composition, lipid peroxidase, dehydroascorbate reductase, catalase, and glutathione reductase enzymes accumulation in barley (Baltruschat et al., 2008). In another instance, the endophytic fungus *Exopiala* sp. LHL08 isolated from cucumber root appeared to confer tolerance against salinity and drought stress in rice (Murphy et al., 2018). Similarly, *P. indica* and *S. vermifera* showed salinity tolerance with increased growth and yield, and strong effects were observed by combining biological agents (Franken, 2012). Endophytic fungi *Fusarium culmorum* (FcRed1) was found to have a symbiotic relationship with rice seedlings under salinity and drought, resulting in increased plant height, root length, biomass, and higher osmolyte accumulation than the non-symbiotic rice seedlings. Nonetheless, there is a great need to unveil how fungal endophytes stimulate plant defense response against abiotic stress (Egamberdieva et al., 2017; Ma et al., 2019).

### High Temperature

Endophytic fungi may mediate the effect of high temperature by adjusting, regulating, or modifying plant physiological, biochemical, and metabolic activity depending on their biology, developmental stage, and cultivar of the host plant. Environmental conditions that affect the endophytic community may also escalate in the number and variety of endophytic populations influencing metabolite production. Many researchers have assayed several isolated fungal endophytes, their multifarious interactions beneficial for the host against high-temperature stress environments, and their precise roles in environment acclimatization and conferring tolerance (Lata et al., 2018). For instance, a study found *Curvularia protuberate*, a fungal endophyte, confer thermo-tolerance to tomato and panic grass. *Paraphaeosphaeri aquadriseptata* produces a heat shock protein 90 (HSP90) inhibitor conferring heat tolerance to Arabidopsis sp. When infected by a virus, as exemplified in a study of the potential of *Stenotrophomonas maltophilia* SBP-9 to promote the growth of wheat under salt stress plant, the plant may confer heat tolerance (Marquez et al., 2007; Yadav and Yadav, 2017). Thus, a critical evaluation of a more intriguing and inexplicable issue with many fungal endophytes to produce host metabolites needed to be harnessed for potential use in a diverse environment (Khare et al., 2018; Galani et al., 2022).

### Heavy Metal Stress

Heavy metal pollution is of great concern in developing countries where industrial wastes and pollutants are directly dumped into rivers or left open on soil surfaces. Cultivable areas may get affected by the application of excessive agrochemicals and polluted water as the direct passage from waterways. Accumulation of such heavy metals in the plants through the soil and irrigated water, hampering their growth and giving direct entry into the food chain, may pose health hazards to humans and animals; hence, bioremediation is crucial. In this context, plant endophyte symbiotic interactions enhance antioxidant enzyme activity *via* uptake of heavy metal (cadmium) from soil followed by detoxification and transport led by three genes, namely ZYP, PCS, and MTP (Ma et al., 2016; Wang et al., 2016). Various fungal endophytes (*Penicillium* sp., *Mucor* sp., *Alternaria* sp., *Microsphaeropsis* sp., etc.) have ameliorated the adverse effects of heavy metal stress in plants. Fungal endophytes are found to be involved in the biosorption of cadmium (Xiao et al., 2010), copper (Li et al., 2012), and sodium chloride (Khan et al., 2013) by stabilizing the negative effect of heavy metal toxicity (Khan et al., 2013). Zahoor et al. (2017) isolated and screened some innate endophytes and cultured them on heavy metal (chromium, cobalt, copper, manganese, and zinc) contaminated soils and found *Mucor* sp. (strain MHR-7) to confer a multiple heavy metal toxicity tolerance. Recently, the endophyte mediated biosensor concept is also receiving much attention, promising a better bioremediation-controlled process (Khanam et al., 2020). Some endophytes induce phytohormone production, especially gibberellins, which contribute to fungus in eradicating of heavy metal toxicity, thus serving an ecofriendly approach to bioremediation benefiting a healthy diet environment.

### Modulation of Plant Defense Mechanism Against Abiotic Stress

It has been hypothesized that fungal microbiota composition alters when plants experience environmental stress, which is determined by the plant genotype (Andreo-Jimenez et al., 2019). Consequently, it takes complementary microbial and plant genes together to establish a symbiotic genetic pathway as a consequence of molecules produced by endophytes that function as signaling molecules within plants, referred to collectively as “symbiont-associated molecular patterns” (SAMPs) (Harman and Uphoff, 2019). Earlier Schulz et al. (1999) proposed the “balanced antagonism” hypothesis in biotic stress resistance, which propounded a balance between plant defensive responsive genes, endophytes, and their toxic effects on each other. Since all the interactions between endophytes and plants are mediated *via* chemical signaling led by gene action, it is essential to understand the mechanisms of how these endophyte function in plants and intensifies the defense system (Chagas et al., 2018; Etalo et al., 2018; Yan et al., 2019). As per the mechanism studied, the fungal spore germ tube initially is triggered by 5-deoxy-strigol under stress, resulting in the branching of hyphae which later spreads and results in colonization accompanied by signaling molecules that lead to induction of plant defense genes triggering phytohormones accumulation. Chemoattractants like flavonoids are the metabolites that assist in the interaction of endophytes with root hair resulting in effective colonization (Khare et al., 2018). Researchers have found signaling molecules such as nod factor, strigolactone, arabinogalactan proteins, plant cell wall proteins, lower levels of ROS (Ca^2+^, NO_2_, and NO), systemin and inositol phosphates, etc., to stimulate symbiotic signaling pathways and delayed induction of jasmonic acid, ethylene, and salicylic acid signaling pathway (Plett and Martin, 2018; Rozpadek et al., 2018). According to the studies, drought condition exerts osmotic stress, whereas salinity stress exerts both ionic and osmotic stress, which is counteracted by the accumulation of abscisic acid (Lata et al., 2018). Mitogen-activated protein kinase (MAPK) and heat shock proteins (HSP) play a crucial role in drought and heat stress, respectively (Lata et al., 2018). Modulation of the plant immune system by endophytes and how they target hormonal response pathways *via* miRNAs has been reviewed in detail by Khare et al. (2018). Plant triggers comparatively transient defense reaction to abiotic stresses generating ROS (Keswani et al., 2019a), and in turn, endophytes produce enzymes for their defense, aiding in modulating and intensifying the plant defense system (Mane et al., 2018). Intensive research on fungal endophytes and how exactly they get influenced by altered climatic conditions prompting the production of such allelochemicals that ultimately trigger the defense mechanism and how this affects plant performance is a requisite.

## Fungal Endophytes for Biotic Stress Management

Pesticides/agrochemicals resistance development in pests and pathogens demands more sustainable solutions, preferably pesticide environmental stewardship under biocontrol agents (Baron and Rigobelo, 2021). In this regard, non-pathogenic fungal endophytes isolates have demonstrated their ability to help other plants withstand subsequent pathogen attacks (Mane et al., 2018; Grabka et al., 2022). They usher upregulation of bioactive molecules, phenolic compounds, antioxidants, phytoalexins, phytohormone production, ecological occupancy, etc. They are responsible for safeguarding crops from biotic stress (Singh et al., 2021). *Aspergillus* sp., *Colletotrichum* sp., *Gliocladium* sp., *Fusarium* sp., *Petriella* sp., *Piriformospora* sp., *Trichoderma* sp., *Epicoccum* sp., *Epichloe* sp., etc., are a few of the major endophytes launching biotic stress resistance, while boosting growth and yield components of the host plant synchronously (Table 3). These major fungal endophytes can potentially accommodate crops in an eco-friendly manner worthy of being treated as biocontrol agents (Adeleke et al., 2022). *Epichloe* sp. offers biotic resistance to its host plant and controls herbivorous insects (Laihonen et al., 2022). *Piriformospora indica*, a root colonizing filamentous fungus belonging to the Basidiomycota class, reside in association with xerophytic plants specifically, found to have a broad host range including most angiosperms viz. wheat, rice, maize, barley, tomato, chili (Arora et al., 2022), etc. It counter-attacks pest/pathogen by accelerating the production of phytoalexins, anti-microbial compounds, activation of antioxidant defense enzyme (glutathione-ascorbate) system, phytohormone production, etc., resistance (Waller et al., 2005). Majorly three counteractions against pests/pathogens are reported, (a) antibiosis (antibiotic production), (b) competition for nutrients (c) mycoparasitism. Wilts, rots, mildews, leaf mosaics, nematodes, blights, etc., can efficiently be controlled by *P. indica;* therefore, they are worthy of being considered a potential biocontrol agent (Waller et al., 2005; Ali et al., 2019). An anamorphic filamentous saprophytic fungus, *Trichoderma* sp. colonizes roots, twigs, and stems of host plants serving as potential biocontrol agents producing anti-fungal, anti-bacterial, and cytotoxic effects. Moreover, they grow naturally in different habitats in varied climatic conditions and easily adapt to edaphic conditions. *Trichoderma* sp. is a universal soil resident and inhabits roots and root hairs of various plants, where it secrets extracellular and intracellular metabolites with cytotoxic effects against fungal pathogens (Srivastava et al., 2012, 2014). Various fungal isolates like *T. harzianum, T. koningii, T. atroviride, T. virens, T. reesei*, and *T*. *citrinoviride* are reported, and their pathogenesis against pathogens has been demonstrated. Strong counter-attack activity against potent pests proves its suitable candidature for bio-control agents (Singh et al., 2021). Few of its strains are already being used as biofertilizers, reducing chemical fertilizer usage on farms. Additionally, *Trichoderma* sp. can produce phytohormones (indole-3-acetic acid (IAA) or auxin analogs, abscisic acid, and gibberellin), siderophores, and other plant growth-promoting substances (e.g., 6-pentyl-αpyrone, cyclonerodiol, harzianolide, and koninginins) enriching soil microflora and aiding soil fertility as well (El Enshasy et al., 2020; Keswani et al., 2020). A new strain, *Trichoderma* phayaoense, has recently been discovered with parasitic and anti-fungal properties against fungal pathogens with 91% mycelial reduction (Nuangmek et al., 2021). *T. botryosum*, *T. caeruloviride*, *T. lentissimum*, and *T. pseudopyramidale* isolated from wild coffee in Africa were reported to render biological control *via* mycoparasitism (Rodríguez et al., 2021). Highly efficient Trichoderma sp. have been recently cultured *via* various methods that produce high xylanase amounts (Ambehabati et al., 2020). Another soil-borne ascomycete fungal endophyte with antagonist property residing in many host plants is *Myrothecium verrucaria*. It is considered a natural mycoherbicide due to its high phytotoxin and lytic enzyme production (Clarke et al., 2007). *Purpureocillium lilacinum* (formerly *Paecilomyces lilacinus*) fungus can colonize root knot nematode (particularly Meloidogyne incognita) and destroys the females, cysts and eggs (Sharma et al., 2020). Similarly, *Aureubasidium pullulans* and *Candida orthopsilosi*s isolated from teak plant can fight against *Alternaria* sp. Causing post harvest disease in citrus (Sukmawati et al., 2021). Generally, antagonistic activities of fungal endophytes are coupled with the synthesis of bioactive defense-related compounds as well as bioactive natural substances (Rustamova et al., 2022), such as anti-microbial, anti-fungal, and anti-viral metabolites, for instance, perfumoid, phomoenamide, dimethyl disulfide, dibenzofuran, methanethiol, ketones, b-1,3- glucan-, b-1,4- glucan-, and b-glucoside-degrading enzymes, Chitin, and β-glucans, β-1, 3 glucanases, chitinase, cellulose, protease, hydrolyzing enzymes, fumonisin, Beauvericin, etc., that contribute to control of pathogens (see Table 2). Many shreds of evidence strongly supporting the potentiality of such fungal endophytes with broad-spectrum pathogenesis and host adaptability mitigating biotic stress in many agriculturally important crops have already been published. Many of them can be utilized as safe, eco-friendly, and effective biocontrol agents for different crop species.

**TABLE 3:** Recently reported fungal endophytes mediated biotic stress management in agricultural crops worldwide.

Crop	Endophytic fungi/Biocontrol agent	Disease/Causal organism	Bioactive compounds	Activity	References
Soybean	*Trichoderma longibrachiatum*, *T. asperellum*, and *T. atroviride*	Root rot/*Rhizoctonia solani*	Pectinase, chitinase, siderophore, IAA	Disease severity controlled by 55–65%	Sallam et al., 2021
Musk melon	*Trichoderma phayaoense*	Gummy stem blight, Wilt/*Stagonosporopsis cucurbitacearum* and *Fusarium equiseti*	Parasitism and anti-fungal compound	81–91% mycelial growth inhibition	Nuangmek et al., 2021
Wheat	*Sarocladium strictum*, *Anthracocystis floculossa*, *Anthracocystis floculossa*, and *Penicillium olsonii*	Fusarium head blight (FHB)/*F. graminearum*	Not studied	70% reduction in FHB	Rojas et al., 2020
Melons	*Trichoderma harzianum, Trichoderma lentiforme, Epicoccum purpurascens*	Rots, collapse, and Wilt/Macrophomina phaseolina, Monosporascus cannonballus, Fusarium sp.	Microbial antagonist, secondary compounds	42–93% inhibition in radial growth	Gonzalez et al., 2020
Tomato	*Beauveria bassiana*	Whitefly/*Bemisia tabaci*	Chitin and β-glucans	Non-prefernce of inocultaed plant	Wei et al., 2020
Cucumber	*Penicillium* sp. and *Hypocrea* sp.	Wilt/*Fusarium oxysporum* f. sp. *cucumerinum*	Anti-fungal agents	Antagonistic effect and 66% mycelial inhibition	Abro et al., 2019
Ginseng	*Trichoderma citrinoviride*	*Botrytis cinerea*, and *Cylindrocarpon destructans*	b-1,3- glucan-, b-1,4- glucan-, and b-glucoside-degrading enzymes	Mycoparasitism, antibiosis and inhibitory effects	Park et al., 2019
Cotton	*Beauveria bassiana*	Cotton leafworm larvae/*Spodoptera littoralis*	Beauvericin	57% larval death	Sánchez-Rodríguez et al., 2018
Tomato	*Beauveria bassiana*	*Fusarium oxysporum* f. sp. *lycopersici* race 3	Lytic enzyme	72% growth inhibition	Culebro-Ricaldi et al., 2017
Chinese Ginseng	*Trichoderma gamsii*	*Scytalidium lignicola, Fusarium flocciferum, Phoma herbarum*, and *Epicocum nigrum*	Volatile organic compound (dimethyl disulfide, dibenzofuran, methanethiol, ketones)	Hyperosmolar, mycoparasitism and antagonistic activity	Chen et al., 2016
Maize	*Cladosporium oxysporum* and *Rigidoporus vinctus*	Maize stem borer/*Chilo partellus*	mycotoxin (fumonisin)	10% mortality rate	Renuka and Ramanujam, 2016
Wheat	*Trichoderma hamatum, Penicillium*, and *Paecilomyces lilacinus*	Tan spot/*Pyrenophora tritici-repentis*	Not studied	Growth suppression	Larran et al., 2016
Black pepper	*Ceriporia lacerata Annulohypoxylon nitens Daldinia eschscholzii, Diaporthe sp., Phomopsis* sp., *Fusarium* sp.	Rot fungus/*Phytophthora capsici*	Antibiosis, mycoparasitism and non-volatile metabolites	Mycoparasitic activity	Sreeja et al., 2016
Tomato	*Beauveria bassiana*	Fruit borer/*Helicoverpa armigera*	Not studied	Insecticidal activity	Qayyum et al., 2015

## Future Prospects and Challenges

In place of escalated pollution, indiscriminate use of agrochemicals, population explosion, depleting fertile land, and food imbalances, adjusted researchers’ attention toward an alternative eco-friendly and sustainable solution for food production. Endophytic fungi gained much interest in agriculture, aiding plant growth processes under mutualistic relationships and mitigating various stresses. Various researchers are constantly studying the rhizosphere of diverse crops to understand the colonial pattern, interactions, and associated benefits. This has led to new fungal endophytes discovery with a broad range of host alliances in extreme environmental conditions or habitats. Applying endophytic fungi as biocontrol agents or biofertilizers, either alone or as a component of integrated pest management packages, may prove a remedial alternative against agrochemical application dependency. The establishment of consortia constituting potential strains of endophytes from local areas of agricultural farms producing complex biocontrol products can be the first farmer-friendly and eco-friendly step toward sustainability. Research and development associated with novel endophyte or endophytic consortiums associated with diverse crops and habitats can play a significant and lasting role in sustenance. Scientific research should focus on genetically modified endophytes by combining two endophytes performing different ecological roles. Optimization of microbial functions to enhance crop production and protection is also required. Detailed fungal community and endomicrobiome study utilizing biotechnological tools (omics) unveiling endophyte-host interaction and molecular patterns. Advance investigation on general and specific host fungal endophytes is required to develop a thriving bio-inoculum leading on the way to organic food crops for a better tomorrow. Hence, bioprospecting studies to isolate such endophytic symbiont fungi and screening them to be directly used in the field is of great importance. Their incorporation into cropping systems through seed treatments, foliar applications, or other means may improve agricultural efficiency and, at the same time, achieve positive environmental impacts (Harman and Uphoff, 2019; Omomowo and Babalola, 2019). The prime challenge is to comprehend its adverse effects on the host plant, such as the transmission of toxins in the food chain and if endophytes start acting as latent pathogens when inoculated artificially on another crop. What if they reach edible portions like fruits, tubers, storage roots, forage, etc.? Secondly, knowledge of the interplay between newly introduced endophyte and native plant endobiome. Its importance can be understood by the fact that endophytes produce antibiotics only in the presence of other endophytes and not in the pure culture (Schulz et al., 2015). Contrarily, some introduced endophytes might eliminate natural endophytes while colonization or form disease on the new host; this propels researchers to scrutinize endophytes and their pathogenesis both beforehand their release as biofertilizers or biocontrol agents (Keswani et al., 2019b). Lack of substantial information about field performance of introduced endophyte and alteration in functioning owing to climatic changes are yet other related aspects to work on. The potential barriers limiting large endophyte scale commercial incorporation are the poor understanding of general and specific plant-endophyte interactions, physio-chemical barriers suppressing the plant immune response stimulation, pathogen-plant immune defense system, gene action, and signaling pathway in agriculture (Ma et al., 2016, 2019). Finally, crop breeding programs have not considered endophytic biodiversity (a breeding objective). There is no report of any marker designed/synthesized favoring endophyte association with crops. Finally, obtaining registration of the bio-formulated or bioagent products before their market placement is virtually a daunting task/challenge. The licensing laws and its regulatory bodies, framing rules and regulations to register and commercialize any product preceding release in the environment, are a time-consuming process. Nevertheless, such regulations ensure in-depth explorative and comprehensive research and understanding to steer clear of ecological concerns eventually. Eminent scientists from different fields are contributing and hopefully succeeding in generating novel solutions for fungal endophytes inoculations/formulations at a commercial scale, ultimately facilitating climate-smart agriculture-led sustainable agriculture.

## Conclusion

It is concluded that crops naturally accommodate diverse communities of symbiotic fungal endophytes, which provide various benefits, including abiotic and biotic resistance leading to high food production. Fungal endophytes are a potentially vital source of metabolites or bioactive compounds for sustained agriculture with significant potential to combat crop pathogens that are becoming increasingly resistant to pesticides. Some of these endophytes can’t be cultured and hence need a more advanced approach to harness the benefits *via* artificial inoculation/bioagents in cultivable crops. However, we need to discover efficient endophytes for climate-resilient and sustainable agriculture to understand their ecological roles and microbial interactions better. Overall fungal endophytes have vast potential to deal with changing climate and plant protection in an eco-friendly manner.

## Author Contributions

HJ contributed to conceptualization and supervision. AV and NS contributed to writing. JP, ES, PP, and RS contributed to writing – review and editing. PP contributed to fund acquisition. All authors contributed to the article and approved the submitted version.

## Conflict of Interest

The authors declare that the research was conducted in the absence of any commercial or financial relationships that could be construed as a potential conflict of interest.

## Publisher’s Note

All claims expressed in this article are solely those of the authors and do not necessarily represent those of their affiliated organizations, or those of the publisher, the editors and the reviewers. Any product that may be evaluated in this article, or claim that may be made by its manufacturer, is not guaranteed or endorsed by the publisher.
